# Breaking down physical barriers: strategies to improve lymphocyte infiltration for effective neoantigen-based therapies

**DOI:** 10.3389/fimmu.2025.1614228

**Published:** 2025-06-12

**Authors:** Ting-Ting Chen, Xiong Li, Yi Zhang, Xiao-Juan Kang, Shu-Fang Zhang, Tong Zhang, Deji Sangmao, Ya-Juan Zhu, De-Kui Zhang

**Affiliations:** ^1^ Department of Gastroenterology, The Second Hospital and Clinical Medical School, Lanzhou University, Lanzhou, China; ^2^ Department of Gastroenterology, The Second Affiliated Hospital of Xi’an Jiaotong University, Xi’an, China; ^3^ The First Clinical Medical College of Lanzhou University, Lanzhou, China; ^4^ Department of Biotherapy and Cancer Center, State Key Laboratory of Biotherapy, West China Hospital, Sichuan University, Sichuan, Chengdu, China

**Keywords:** neoantigen, tumor-infiltrating lymphocytes, extracellular matrix, interstitial fluid pressure, cancer-associated fibroblasts

## Abstract

The cancer genomic instability drives the generation of neoantigens, making them ideal targets for immunotherapy. Neoantigen-specific tumor-infiltrating lymphocytes achieve precise tumor cell killing by recognizing neoantigens on the tumor surface, but their efficacy is limited by complex physical barriers within the tumor microenvironment. These barriers not only directly impede TIL migration and infiltration but also synergize with immunosuppressive signals to weaken antitumor immune responses. The tumor extracellular matrix forms a dense fibrous network due to enhanced collagen crosslinking, pathological hyaluronic acid deposition, and increased stiffness, hindering TIL mobility. Aberrant tumor vasculature, characterized by hyperpermeability and elevated interstitial fluid pressure, collaborates with pro-fibrotic factors, such as VEGF, TGF-β secreted by cancer-associated fibroblasts and regulatory T cells to create mechanical compression barriers. This review systematically explores the composition, molecular mechanisms, and therapeutic strategies targeting these physical barriers, providing novel insights for neoantigen-based therapies. Future efforts should integrate biomechanical interventions with immunotherapy, elucidate the interplay between mechanical signaling and immunometabolism, and optimize multi-target combinatorial approaches to enhance the clinical translation potential of neoantigen therapies.

## 1 Introduction

Cancer is a leading cause of death worldwide. The genetic instability of tumor cells not only correlates with metastasis, therapy resistance, and immune evasion, but also accelerates tumor evolution through the accumulation of mutations. This process enhances the genetic diversity of tumor cells, ultimately resulting in the formation of highly heterogeneous cell populations (1, 2). It is noteworthy that the genetic instability of tumor cells can also lead to a high burden of mutations. Proteins or peptide sequences derived from nonsynonymous mutations, which are exploited in cancer therapy, are termed neoantigens (3). Neoantigens are antigens derived from mutated proteins and can also be generated through mechanisms such as viral infection, alternative splicing, and gene rearrangement. These antigens are predominantly overexpressed in tumor cells, exhibiting high immunogenicity and significant tumor heterogeneity (4). Neoantigen vaccines have demonstrated significant efficacy in clinical trials for cancer treatment. Tumor neoantigens serve as ideal targets for lymphocyte recognition, and their application can stimulate robust anti-tumor immune responses by promoting the generation of tumor-infiltrating lymphocytes (TILs) (4).

Current systemic therapeutic approaches for cancer include chemotherapy, hormone therapy, targeted therapy, and immunotherapy. Patients with higher levels of TILs generally demonstrate improved therapeutic efficacy and prognosis with these treatments (5). Beyond their role as prognostic biomarkers, the presence of TILs has also been shown to predict sensitivity to immunotherapy, chemotherapy, and other targeted therapies (6, 7). In therapeutic applications, TIL-based therapy represents a cutting-edge approach in personalized cancer treatment. The TIL therapy protocol—involving surgical resection of tumor specimens, isolation of infiltrating lymphocytes, ex vivo expansion, and reinfusion into patients—has demonstrated promising clinical outcomes in solid malignancies such as melanoma and cervical cancer (8, 9).

Neoantigen-specific TILs have garnered significant attention in cancer immunotherapy. The “neoantigen-targeting specificity” refers to the ability of these TILs to directly recognize neoantigens presented on tumor cell surfaces, enabling precise tumor cell elimination while minimizing off-target damage to healthy tissues (10). Neoantigen-specific TILs mediate anti-tumor immune responses through four critical steps: (1) neoantigen generation and presentation, (2) T-cell activation and clonal expansion, (3) T-cell trafficking to tumor sites, and (4) target recognition and tumor cell killing (11–14). However, the efficacy of such therapies is highly dependent on the immune status of the tumor microenvironment (TME). Based on immune cell infiltration patterns, tumors can be classified into three major phenotypes: immune-desert phenotype, immune-excluded phenotype, and inflamed phenotype (2). Clinically termed “cold tumors,” those with low or absent lymphocyte infiltration often exhibit resistance to neoantigen-based immunotherapies (15). The cytotoxic function of neoantigen-specific TILs is further constrained by the profoundly immunosuppressive nature of the TME. The therapeutic potential of neoantigen-specific TILs is impeded not only by classical immunosuppressive factors—such as infiltration of immunosuppressive cells (e.g., regulatory T cells, myeloid-derived suppressor cells) and accumulation of inhibitory metabolites (e.g., adenosine, lactate)—as highlighted in traditional immunotherapy research (16), but also by physical barriers encountered during TIL activation, migration, and infiltration. These biophysical constraints severely restrict cellular metabolism and functional execution.

In this review, we systematically analyze the physical barriers confronting neoantigen-specific TILs in cancer immunotherapy and discuss potential strategies to overcome these limitations, thereby providing a theoretical framework for enhancing the therapeutic efficacy of neoantigen-specific TIL-based immunotherapies.

## 2 Components of physical barriers of neoantigen-specific lymphocytes

### 2.1 Tumor extracellular matrix

#### 2.1.1 Pathological remodeling features of extracellular matrix

The extracellular matrix (ECM) is a highly dynamic structure continuously remodeled through cellular activities such as synthesis, degradation, reorganization, and chemical modification (17). At the molecular level, the core components of ECM include structural proteins (e.g., collagen, elastin), adhesive proteins (e.g., fibronectin, laminin), polysaccharides (e.g., hyaluronic acid, heparan sulfate proteoglycans), and matrix-remodeling enzymes (e.g., matrix metalloproteinases, lysyl oxidases) (18). Structurally, the ECM primarily comprises the basement membrane, fibrous networks, and hydrated gel-like matrices (19–21). These components collectively form a three-dimensional microenvironment that provides essential biomechanical support and biochemical signaling to maintain tissue architecture and function. The tumor ECM is a critical component of the TME and plays a pivotal role in tumor initiation, progression, metastasis, and therapeutic resistance (22). Compared to normal ECM, the tumor ECM exhibits distinct pathological features, including fibrosis, enhanced crosslinking, and increased tissue stiffness (17). These biomechanical alterations in the tumor ECM represent one of the core physical barriers faced by neoantigen-specific TILs during antitumor immune responses. The tumor ECM and fibrotic stroma form structural physical barriers, leading to the entrapment of TILs in the peritumoral or interstitial regions, resulting in an immune-excluded phenotype (23). The impaired migration of neoantigen-specific TILs and their inability to infiltrate tumor tissues constitute major obstacles to therapies based on neoantigen-specific TILs.

#### 2.1.2 Barrier mechanisms of key ECM components

Collagen, a major component of the tumor ECM, forms a dense mesh-like structure that provides physical support to tumors. The activity of neoantigen-specific TILs against tumor cells is influenced by the arrangement and density of collagen within the ECM. Physically, moderate collagen fiber alignment facilitates TIL migration, whereas dense collagen networks formed by high collagen density reduce the motility of neoantigen-specific TILs and impede T-cell infiltration into the tumor core (24, 25). Gene families involved in collagen post-translational modification, crosslinking, and degradation regulate its physicochemical and immune properties. Lysyl oxidase (LOX) family proteins catalyze the conversion of lysine residues in collagen and elastin precursors into highly reactive aldehyde groups, triggering crosslinking and stabilization of ECM proteins (e.g., type I collagen and elastin) and modulating cell adhesion, migration, and invasion (17). Overexpression of LOX, a collagen crosslinking enzyme, increases matrix stiffness, which is critical for maintaining tissue mechanical strength and regulating matrix rigidity, and is closely associated with T-cell exhaustion (24, 26).

Hyaluronic acid (HA), a key component of the tumor ECM, dynamically regulates the TME through a synthesis-degradation balance. Beyond its inherent properties, HA molecular weight plays a pivotal role in cancer progression. High-molecular-weight HA (HMW-HA) exhibits anti-angiogenic, anti-inflammatory, and immunosuppressive effects but may promote matrix stiffening in certain contexts (e.g., pancreatic cancer). Low-molecular-weight HA (LMW-HA) is linked to inflammation, angiogenesis, and tumor progression (27). The specific roles of HA molecular weight require further investigation. HA itself forms a physical barrier that hinders TIL infiltration and reduces the efficacy of neoantigen-based therapies. Cancer-associated fibroblasts (CAFs) secrete HA to remodel the ECM, while HA reciprocally regulates fibroblast proliferation, directly creating a physical barrier that impedes neoantigen-specific TIL activity. Despite the barrier effect of HA, its dual biological roles in cancer therapy must be acknowledged. Therapeutic strategies targeting HA demand deeper exploration, integrating molecular weight specificity, tumor type, and microenvironment characteristics to optimize HA-targeted drug applications.

During tumor progression, the normal ECM is degraded and replaced by tumor-specific ECM. Tumor-driven ECM remodeling involves mechanical forces exerted by tumor cells, leading to nonlinear stiffening and plastic deformation of the ECM, which significantly alters its microstructure. Tumor cells and CAFs also secrete matrix metalloproteinases (MMPs) to degrade the ECM, compromising its structural integrity (28). Consequently, the remodeled tumor ECM exhibits higher density and stiffness due to abnormal deposition of collagen, HA, and other components. This dense and rigid ECM acts as a mechanical barrier to neoantigen-specific TILs (24). Fibrotic regions of the tumor ECM create a compact and stiff microenvironment that restricts the infiltration and migration of neoantigen-specific TILs (29).

**Figure 1:**
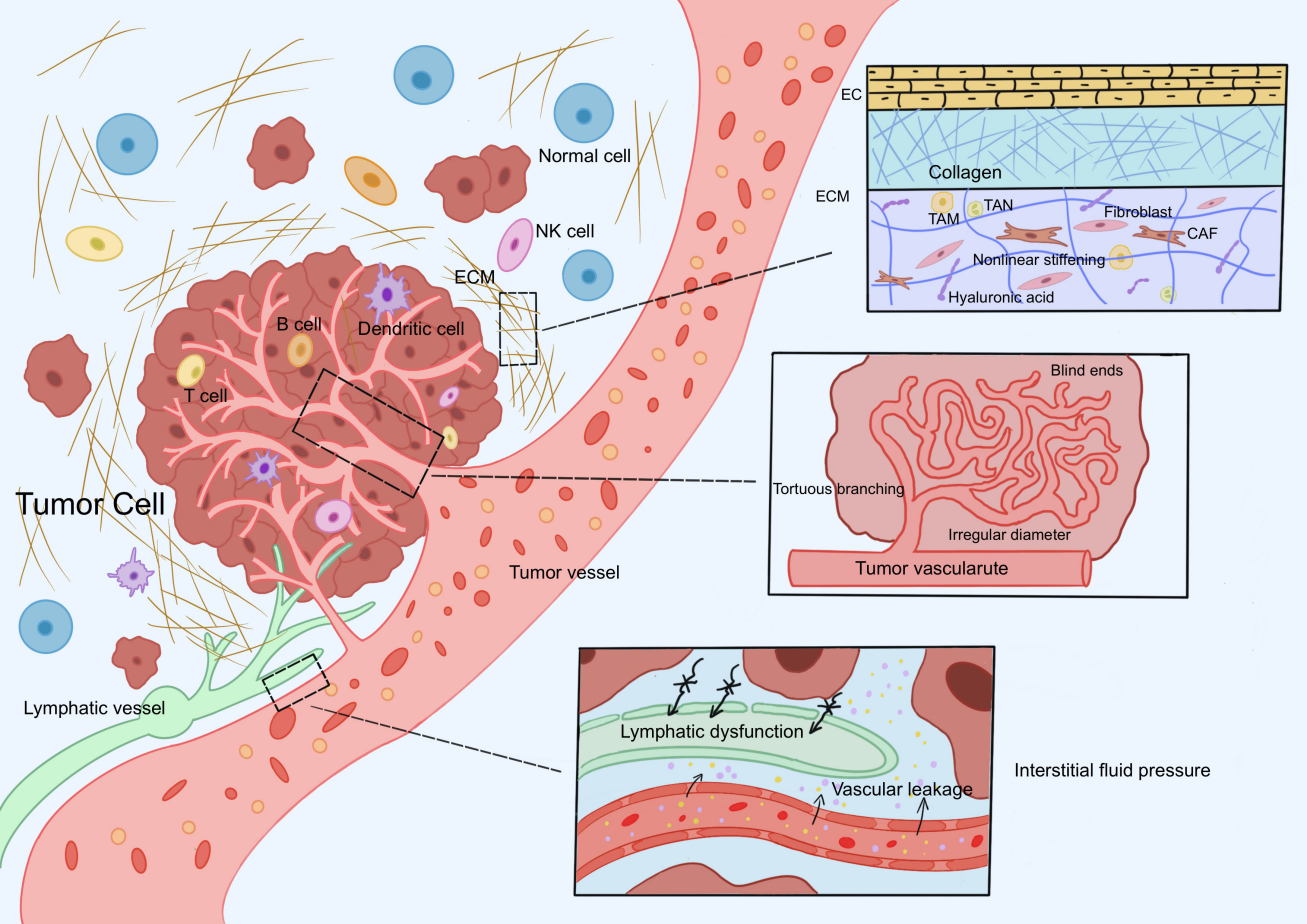
Physical barrier mechanisms of neoantigen-specific TILs. Neoantigen-specific TILs encounter three core physical barriers during activation, migration, and infiltration: abnormal ECM, dysregulated tumor vasculature, and elevated IFP. The tumor ECM forms a dense three-dimensional network due to fibrosis, increased crosslinking density, and elevated tissue stiffness. Tumor vasculature exhibits uneven luminal diameters, distorted morphology (e.g., blind-end structures), loosely arranged endothelial cells, and abnormal connections between the basement membrane and pericytes. High IFP results from the synergistic effects of abnormal vascular leakage, impaired lymphatic drainage, and excessive ECM deposition. Abbreviation: TILs: tumor-infiltrating lymphocytes, ECM: extracellular matrix, IFP: interstitial fluid pressure.

The abnormal deposition of tumor ECM influences neoantigen-specific TILs behavior and function through multiple mechanisms. Notably, while the rigid fibrotic ECM directly impedes TIL infiltration into the tumor core, it also synergizes with biochemical signals to suppress TIL activity. Studies indicate that Osr2 is specifically upregulated in CD8+ T cells within high-stiffness ECM regions of the tumor microenvironment. Its expression depends on the synergistic effects of TCR signaling and mechanical stress from the tumor ECM. High-stiffness ECM activates the mechanosensitive ion channel Piezo1, triggering Ca²^+^ influx, activating the CaMKII/CREB signaling pathway, inducing Osr2 expression, and driving T-cell exhaustion. Inhibiting Osr2 may reverse T-cell exhaustion and enhance the efficacy of neoantigen-specific TILs against tumors (30). Therefore, combined therapeutic strategies targeting both the physical ECM barrier and biochemical signaling pathways may improve neoantigen-specific TIL functionality and enhance the effectiveness of tumor neoantigen therapies (**Figure 1**).

### 2.2 Abnormal tumor angiogenesis

#### 2.2.1 Structural and functional abnormalities of tumor vasculature

The structural and functional abnormalities of the tumor vascular system are critical factors limiting the efficacy of neoantigen-specific TILs. Normal vasculature exhibits a hierarchical structure, branching from large veins or arteries into smaller capillary networks interconnected by delicate micro vessels to ensure efficient nutrient and oxygen exchange. In contrast, tumor vasculature is characterized by structural and functional defects. Structurally, tumor vessels display irregular luminal diameters, tortuous and blind-ended shapes, and heterogeneous density (31, 32). These vessels are composed of endothelial cells, mural cells (e.g., pericytes), and a surrounding basement membrane (33). However, endothelial cells are loosely arranged with reduced intercellular junctions, and the basement membrane is fragmented and poorly connected to endothelial cells and pericytes (34). These structural defects result in high endothelial proliferation, hyperpermeability, chaotic blood flow, loss of hierarchical organization, and insufficient pericyte coverage (35, 36), all of which directly impede the penetration of neoantigen-specific TILs through the vascular wall.

#### 2.2.2 Molecular mechanisms of angiogenic imbalance

At the molecular level, angiogenesis is regulated by a dynamic balance between pro-angiogenic factors and endogenous angiogenesis inhibitors (37). VEGF, particularly VEGF-A, is a key regulator of tumor angiogenesis, driving both physiological and pathological vascular growth (38). Beyond endothelial cells, multiple cell types in the tumor microenvironment, including endothelial progenitor cells, lymphatic endothelial cells, pericytes, and vascular smooth muscle cells, respond to VEGF signaling (37). The canonical VEGF pathway involves VEGF binding to VEGFR-2, activating downstream PI3K-AKT, MAPK/ERK, and NF-κB pathways to induce endothelial cell proliferation, migration, and lumen formation. Overproduction of VEGF increases vascular permeability, leading to plasma protein leakage and elevated IFP, which hinders the migration of neoantigen-specific TILs (39). Fibroblast growth factor (FGF), another well-known angiogenic factor stored in the vascular basement membrane, also contributes to vascular development and progression via its signaling pathways (40). Fibroblast growth factor-2 (FGF-2), a member of the FGF family (also known as basic FGF), exhibits potent angiogenic activity comparable to VEGF-A (38).

#### 2.2.3 Synergistic role of tumor ECM component HA in vascular abnormalities

HA, a key tumor ECM component discussed earlier, promotes tumor angiogenesis through multiple mechanisms. HA accumulation in tumors enhances the recruitment of monocytes and macrophages, which secrete pro-angiogenic factors like VEGF to directly stimulate neovascularization. Increased hyaluronidase (HYAL) activity and reactive oxygen species (ROS) production in tumors degrade high-molecular-weight HA (HMW-HA) into low-molecular-weight HA (LMW-HA). LMW-HA fragments activate specific HA-binding proteins (e.g., RHAMM), inducing endothelial cell actin cytoskeleton reorganization and disrupting intercellular junctions (e.g., VE-cadherin). This reduces vascular integrity, allowing leaky vessels to release pro-angiogenic signals, forming a positive feedback loop that accelerates tumor angiogenesis (41).

#### 2.2.4 Synergistic effects of multiple barriers

Beyond directly forming a vascular barrier that hinders neoantigen-specific TIL penetration, abnormal tumor vasculature interacts with the tumor ECM to create a combined physical barrier, further limiting TIL infiltration into tumors (42). Additionally, the hyperpermeability of tumor vessels elevates interstitial fluid pressure, generating a compressive physical barrier that restricts the mobility and efficacy of neoantigen-specific TILs, thereby constraining therapeutic outcomes (43, 44).

### 2.3 Elevated interstitial fluid pressure

Elevated Interstitial Fluid Pressure (IFP) in the TME is a critical physical barrier that impedes the infiltration and function of neoantigen-specific TILs. High IFP results from multiple mechanisms, including vascular leakage and dysfunctional lymphatic drainage. First, the abnormal tumor vasculature described earlier exhibits hyperpermeability and leakage, allowing plasma proteins and fluid to extravasate into the interstitium. This increases interstitial fluid volume, elevates IFP, and creates a mechanical barrier that hinders neoantigen-specific TIL infiltration (45, 46). Notably, this pressure environment interacts bidirectionally with the vascular system: aberrant tumor vasculature contributes to IFP, while IFP conversely compresses blood vessels, limiting the extravasation of neoantigen-specific TILs into tumor tissue (47). Second, discontinuous basement membranes and a lack of pericyte coverage in tumor lymphatic vessels result in high permeability and low shear stress, leading to dysfunctional lymphatic drainage and elevated IFP (48, 49). The interplay between lymphatic dysfunction and vascular leakage forms a vicious cycle, causing persistent interstitial fluid accumulation and progressive IFP elevation, which physically compresses and restricts TIL infiltration (50). Excessive ECM deposition further restricts interstitial fluid flow, exacerbating fluid accumulation within the tumor (51). The role of HA in contributing to IFP remains debated. Local injection of hyaluronidase into osteosarcoma xenografts has been shown to reduce IFP, whereas forced overexpression of HA in thyroid and colon cancer xenografts does not increase IFP (52–54). Beyond its physical compressive effects, elevated IFP promotes interstitial fluid flow, exposing neoantigen-specific TILs to shear stress. Shear stress significantly impacts the biological behavior of TILs in multiple ways, including activating CAFs, influencing tumor angiogenesis and lymphangiogenesis, and inducing matrix metalloproteinase (MMP) activation. These effects are primarily mediated by mechanosignal transduction through focal adhesions, glycocalyx, cell-cell junctions, ion channels, and Notch receptors. Such mechanical signaling can upregulate transforming growth factor-β (TGF-β) expression and activate the YAP/TAZ pathway (51). Under the combined regulation of these factors, ECM remodeling and abnormal vasculature formation are induced, ultimately leading to impaired TIL infiltration and functional suppression.

### 2.4 Functional roles of key cellular components

#### 2.4.1 Fibroblasts

Fibroblasts regulate the structure and function of normal tissues by synthesizing the ECM and facilitating tissue repair. CAFs are perpetually activated fibroblasts within the tumor microenvironment. As key players in shaping the biophysical properties of the tumor microenvironment, CAFs deposit abundant ECM fibers (including collagen) and crosslinking enzymes such as LOX, leading to increased matrix stiffness and fibrous reorganization. These processes create a compact physical barrier that impedes the activity of neoantigen-specific TILs (55). Fibroblasts are the primary source of collagen and are responsible for organizing and aligning collagen fibers (56). In a TGF-β-dependent manner, CAFs not only influence ECM remodeling but also contribute to HA production, which participates in ECM remodeling by generating high IFP (57). Fibroblast activation protein (FAP), a specific marker of CAFs, exhibits unique endopeptidase and exopeptidase activities by cleaving after proline residues. FAP processes ECM proteins to promote tissue remodeling and activates growth factors and cytokines, including TGF-β, thereby enhancing fibroblast activation and immune suppression (58). Overexpression of FAP may lead to ECM fragment accumulation and the formation of dense, disorganized fibrous networks. Additionally, FAP synergizes with the TGF-β/Smad pathway to further amplify CAF activation and ECM synthesis (59). Increased CAF abundance and overexpression of growth factors such as platelet-derived growth factor (PDGF), TGF-β, and fibroblast growth factor 2 (FGF2) are closely associated with tumor angiogenesis and ECM remodeling (60).

#### 2.4.2 Regulatory T cells

Regulatory T cells (Tregs) are a subset of CD4+ T cells with immunosuppressive properties (61). While direct physical barrier mechanisms of Tregs against neoantigen-specific TILs are less studied, previous research has focused on their immunosuppressive functions, such as secreting immunomodulatory cytokines and cytotoxic molecules to suppress TIL activity or modulating antigen-presenting cell function (62, 63). However, the physical obstruction of neoantigen-specific TIL infiltration and function by Tregs should not be overlooked. First, Tregs accumulate densely in tumor tissues, forming high-density zones that physically occupy space and restrict the migration of neoantigen-specific TILs toward the tumor core (64). Second, Tregs promote physical barrier formation by triggering matrix remodeling and abnormal tumor vasculogenesis through multiple mechanisms: Treg-secreted TGF-β induces CAF differentiation, driving collagen crosslinking and fibrin deposition to form dense ECM that hinders neoantigen-specific TIL migration (65); Tregs also secrete VEGF-A and IL-10 to promote immature, leaky vasculature, which impedes effector cell infiltration (66). Furthermore, collagen can upregulate Treg markers such as FoxP3, enhancing Treg differentiation and thereby reinforcing both physical and immune barriers against neoantigen-specific TILs (24).

### 2.5 Dual regulatory role of tumor cell-cell junctions

Cell-cell junctions are crucial structures for maintaining tissue architecture and function. The main types of cell-cell junctions include tight junctions, gap junctions, and adherens junctions. The role of tumor cell-cell junctions in neoantigen-specific TILs is complex, and their intricate regulatory mechanisms determine their “double-edged sword” characteristics in tumors. Tight junctions are primarily composed of transmembrane proteins (e.g., claudins, occludin) and cytoplasmic scaffold proteins (e.g., ZO-1, cingulin), which maintain endothelial barrier integrity, form selective barriers, and regulate permeability (67, 68). In the tumor microenvironment, tumor cells secrete factors such as VEGF, TGF-β1, and ANGPTL4 to downregulate claudin-5 and ZO-1, thereby weakening the endothelial barrier (69). However, abnormally enlarged gaps between tumor vascular endothelial cells may allow macromolecules like plasma proteins to enter the interstitium, further increasing IFP (70). This mechanism may impair the ability of neoantigen-specific TILs to reach tumor sites and exert their effects. Gap junctions, formed by connexin family proteins (Cx37, Cx40, Cx43), regulate intercellular communication, permeability, and coordinated cellular activities. Wang et al. found that Cx43 suppresses VEGF expression in tumor cells, reducing tumor angiogenesis (71). Elizabeth McLachlan et al. also demonstrated that connexin overexpression modulates multiple angiogenesis-related proteins, inhibiting tumor angiogenesis (72). However, beyond these tumor-suppressive effects (e.g., reduced angiogenesis), their pro-tumorigenic roles in certain contexts cannot be overlooked. Studies show that Cx43 is upregulated in hepatocellular carcinoma, enhancing invasiveness and metastasis by forming gap junctions with endothelial cells (73). Additionally, during fibroblast-to-cancer-associated fibroblast transformation, increased gap junction molecule expression strengthens stromal signal transduction, promoting tumor progression (74). Current research highlights the paradoxical role of connexins in cancer development, with their expression linked to both favorable and poor prognoses (75). Adherens junctions are primarily composed of E-cadherin, which maintains tissue mechanical strength (69). In most epithelial-derived malignancies (e.g., breast, gastric, and colorectal cancers), tumor cells typically exhibit downregulated E-cadherin expression. This downregulation is closely associated with tumor invasion, metastasis, and poor prognosis (76–78). E-cadherin may also directly or indirectly participate in forming physical barriers for neoantigen-specific TILs through the Hippo-YAP/TAZ pathway (79). Through dynamic regulation of endothelial barrier integrity, intercellular communication, and mechanical signaling, tumor cell-cell junctions exhibit complex dual roles in the tumor immune microenvironment.

### 2.6 Chemokine-mediated physical barriers

Chemokines are a family of small chemotactic cytokines that play a critical role in regulating the tumor microenvironment, structurally classified into four subfamilies: CXC, CC, XC, and CX3C (80). Key members of the chemokine family, CXCL9, CXCL10, and CXCL11, bind to their shared receptor CXCR3 to regulate immune cell differentiation, directional migration, and tumor infiltration. The CXCL9/10/11–CXCR3 axis guides immune cell chemotaxis toward tumor sites through concentration gradients (81) and antagonizes VEGF to suppress tumor angiogenesis (82). Abnormal chemokine secretion in the tumor microenvironment disrupts these gradients; for example, CCR2/CCL2 and CXCR2/CXCL1 recruit immunosuppressive cells such as myeloid-derived suppressor cells, tumor-associated macrophages, and neutrophils, promoting abnormal vascularization and physically isolating neoantigen-specific TILs (80). Additionally, studies reveal that CXCL12 is highly expressed in high-stromal tumors, while TILs exhibit CXCR4 overexpression. CXCL12 enrichment in stromal regions traps TILs around the stroma via CXCL12-CXCR4 interactions, preventing their infiltration into the tumor core (83).

## 3 Key driving mechanisms of physical barrier formation

### 3.1 TGF-β/Smad signaling axis-mediated ECM remodeling

TGF-β, a prototypical multifunctional cytokine, is a critical regulator of ECM assembly and remodeling. Signaling by TGF-β family members occurs through type I (TβRI) and type II (TβRII) receptors (84). TβRI and TβRII are transmembrane serine/threonine kinases with structural similarities, but the type I receptor contains a conserved glycine/serine-rich (GS box) domain upstream of its kinase domain. Ligand binding induces the assembly of type I and type II receptors into a complex, where TβRII phosphorylates and activates TβRI (85). Signaling from activated TβRI to the nucleus is primarily mediated by the phosphorylation of cytoplasmic protein intermediaries belonging to the Smad family. Smad proteins exhibit competitive pro-fibrotic and anti-fibrotic roles and are involved in fibrosis regulation. Additionally, downstream effectors of the TGF-β/Smad pathway, such as matrix metalloproteinases (MMPs), tissue inhibitors of metalloproteinases (TIMPs), and connective tissue growth factor (CTGF), directly participate in ECM remodeling (86). Excessive activation of TGF-β signaling can lead to abnormal collagen deposition and ECM stiffening, directly forming a physical barrier that impedes TILs infiltration.

### 3.2 Mechanosignaling via the hippo-YAP/TAZ pathway

YAP/TAZ, key transcriptional co-activators in the Hippo signaling pathway, play a significant role in the formation of physical barriers within the TME, directly affecting the functionality and infiltration efficiency of neoantigen-specific TILs. The core downstream effectors of the Hippo pathway are the transcriptional co-activators YAP and its paralog TAZ. When the Hippo pathway is activated, YAP/TAZ are phosphorylated and retained in the cytoplasm or degraded. Unphosphorylated YAP/TAZ translocate to the nucleus, where they bind transcription factors such as TEAD to activate target genes. The Hippo-YAP/TAZ pathway acts as a sensor for the mechanical properties of the extracellular environment, regulated by external cues and stimuli (87). YAP, a potent tumor promoter, is commonly activated during tumor progression. ECM stiffness, cell-cell contact, and fluid shear stress influence YAP/TAZ nuclear localization through Hippo pathway activation or inactivation (88). YAP is critical in converting quiescent fibroblasts into activated fibroblasts across normal and malignant tissues, thereby increasing tumor ECM stiffness (89). Studies show that YAP is highly expressed in Tregs (90). In hepatocellular carcinoma, YAP promotes the differentiation of naïve T cells into Tregs by directly upregulating TβRII expression (91). YAP and TAZ activation in cancer cells also enhances the expression of pro-angiogenic factors like VEGF, driving neovascularization (89). The E-cadherin-mediated cell adhesion enhances LATS1/2-mediated phosphorylation of YAP/TAZ, promoting their retention in the cytoplasm and suppressing nuclear transcriptional activity. The loss of E-cadherin leads to Hippo pathway inactivation, thereby facilitating the formation of physical barriers (79). These findings illustrate how the Hippo-YAP/TAZ pathway integrates physical signals (e.g., ECM stiffness, fluid shear stress) to influence neoantigen-specific TILs through direct and indirect mechanisms.

### 3.3 PDGF/PDGFR pathway-driven stromal remodeling

PDGF ligands and their receptors, PDGFRα and PDGFRβ, play pivotal roles in regulating biological functions such as cell growth, survival, and migration. PDGF-PDGFR interaction induces receptor dimerization and tyrosine phosphorylation, triggering intracellular signaling cascades. The PDGF signaling network comprises four ligands—PDGF-A, PDGF-B, PDGF-C, and PDGF-D—that interact with PDGFRα and PDGFRβ (92). PDGFRα/β drives tumor growth by activating downstream pro-survival pathways like PI3K-AKT and MEK-ERK (93). Additionally, PDGF is a key mediator of cancer-associated stromal fibroblast (CAF) proliferation (94). PDGF activates CAFs via PDGFR binding, inducing the secretion of ECM components such as collagen (types I and III) and fibronectin (95). Studies demonstrate that inhibiting PDGFR signaling in CAFs reduces their replicative capacity and ECM deposition (55). Using a 3D microengineered organotypic tumor-stroma model, Harpinder Saini et al. confirmed that suppressing PDGFR activity in CAFs decreases tumor ECM stiffness (55). Thus, targeting the PDGF/PDGFR pathway may not only weaken physical barriers but also enhance TIL infiltration efficiency by improving TME mechanical properties, and facilitating neoantigen-specific TIL activity.

**Figure 2:**
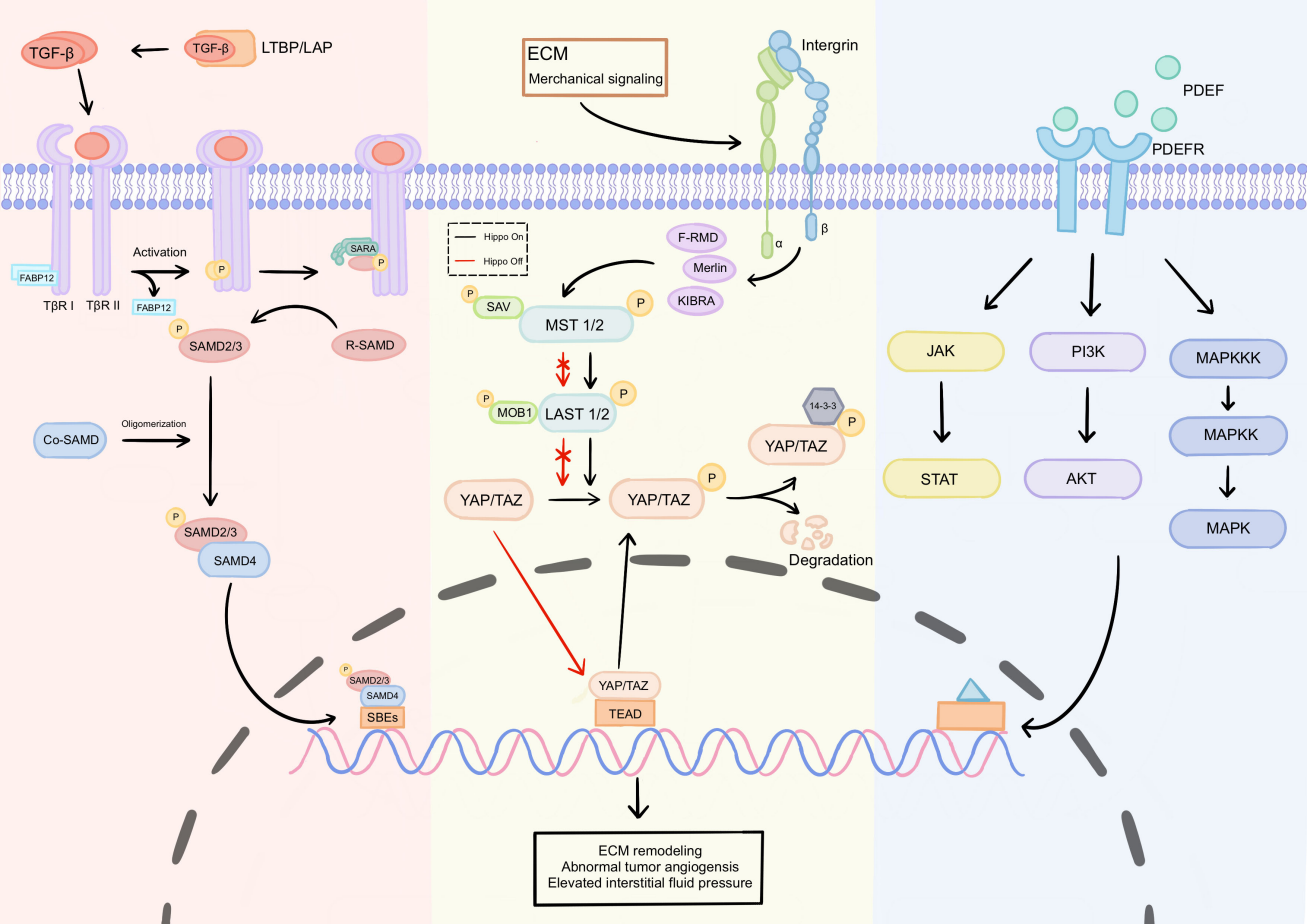
Key signaling pathways driving physical barrier formation. The formation of physical barriers is primarily driven by three core signaling pathways: TGF-β/Smad, Hippo-YAP/TAZ, and PDGF/PDGFR: TGF-β/Smad pathway: Ligand binding to TβRII and TβRI receptors forms a complex, triggering TβRII phosphorylation and subsequent activation of TβRI, which phosphorylates Smad2/3 proteins. Phosphorylated Smad2/3 binds to Smad4 and translocates to the nucleus to regulate target gene expression. Downstream effectors directly mediate ECM remodeling. Hippo-YAP/TAZ pathway: This pathway regulates YAP/TAZ activity by sensing mechanical signals such as ECM stiffness and fluid shear stress. When the Hippo pathway is inactive, unphosphorylated YAP/TAZ enters the nucleus and binds to transcription factors like TEAD, activating pro-fibrotic target genes. This drives fibroblast activation into CAFs and ECM component secretion, increasing matrix stiffness. PDGF/PDGFR pathway: Ligand-receptor binding induces receptor dimerization and tyrosine phosphorylation, activating downstream pathways such as PI3K-AKT, MEK-ERK, and JAK-STAT. These pathways promote tumor growth and CAF activation. ECM, extracellular matrix; CAF, Cancer-associated fibroblast.

The signaling pathways driving physical barrier formation do not act in isolation but collaborate to form an interactive regulatory network. TGF-β directly remodels the ECM via Smad signaling while synergizing with YAP/TAZ to respond to mechanical stress. PDGF-driven CAF activation amplifies abnormal ECM deposition, and YAP-mediated Treg differentiation reinforces barrier effects through immune suppression and physical obstruction. This multidimensional regulation results in a composite TME barrier characterized by both physical resistance and immunosuppression, ultimately hindering the efficacy of neoantigen-specific TILs (**Figure 2**).

## 4 Therapeutic strategies targeting physical barriers

Physical barriers not only act as spatial obstacles to the infiltration of neoantigen-specific TILs but also serve as biomechanical signaling sources driving immunosuppression. The presence of these barriers highlights the limited efficacy of single-agent immunotherapy, necessitating combination with other therapies. Multiscale mechanical interventions can establish a “barrier-breaking” therapeutic system for neoantigen-based therapies by dismantling physical barriers, thereby promoting the migration, infiltration, and functional activity of neoantigen-specific TILs into the tumor core (**Table 1**).

**Table 1:** Classification of therapeutic strategies targeting physical barriers.

Therapeutic strategy	Mechanism of action	Representative agents/technologies	Key targets	Advantages	Challenges
Inhibition of Crosslinking	Block LOX family-mediated collagen crosslinking	β-aminopropionitrile (BAPN) (96)	LOX	Reverses matrix stiffness, improves TIL infiltration and drug penetration	Systemic toxicity
Collagen Suppression	Inhibit TGFβ signaling via attIL12-T, induce CAF apoptosis, reduce collagen deposition	attIL12-T (97)	TGFβ/FAS/caspase-3	Localized immune activation, ECM loosening	Long-term safety requires validation
YAP/TAZ Inhibition	Degrade YAP/TAZ proteins, disrupt YAP/TAZ-TEAD interaction, block nuclear localization	Verteporfin (98, 99), ION537 (100, 101)	YAP/TAZ-TEAD pathway	Suppresses CAF activity, reduces ECM deposition	Off-target effects (e.g., interference with autophagy)
Hyaluronic Acid Degradation	Degrade excessively deposited hyaluronic acid (HA) in ECM	PEGPH20 (102, 103)	Hyaluronic Acid (HA)	Rapidly improves TIL infiltration	May accelerate metastasis and tumor cell spread
Anti-VEGF Therapy	Normalize vascular permeability and perfusion	Bevacizumab (104)	VEGF-A	Enhances drug delivery	Resistance, toxicity, and short-term treatment may increase tumor invasiveness
Angiopoietin Modulation	Stabilize vascular endothelial junctions	Trebananib, AMG-386 (105–107)	Tie2 receptor	Reduces vascular leakage	May disrupt vascular homeostasis in normal tissues

### 4.1 LOX inhibitors

Tumor stiffness is associated with the structure of the ECM. High-stiffness tumors exhibit dense, linearized collagen fibers that form physical barriers, whereas softer tumors have looser collagen fibers and enhanced T-cell migratory capacity (24). LOX-mediated collagen crosslinking is a key mediator of increased tumor stromal stiffness and a driver of metastatic tumor growth. The LOX inhibitor β-aminopropionitrile (BAPN) suppresses LOX enzymatic activity, blocks collagen crosslinking, reverses tumor ECM stiffness, and reduces collagen fiber thickness, linearization, and density. Studies in breast cancer demonstrate that inhibiting LOX and blocking TGF-β reduce collagen network stiffness and density in mammary tissue, suggesting this network as a potential therapeutic target (108). LOX inhibition not only improves neoantigen-specific T-cell migration and infiltration but also enhances drug penetration in anticancer therapies (96).

### 4.2 Collagen suppression

A novel study proposed that membrane-anchored, tumor-targeted IL-12 T cells (attIL12-T) trigger IFNγ release by binding to CSV, inhibit TGF-β signaling, upregulate FAS expression on CAFs, and activate the caspase-3/PARP apoptosis pathway. This leads to loss of collagen contractility, loosened ECM structure, and opening of T-cell infiltration channels (97). By anchoring IL-12 to T-cell membranes, attIL12-T minimizes systemic toxicity while achieving localized immune activation via CSV-mediated tumor targeting. Combining this therapy with neoantigen immunotherapy may enhance efficacy in ECM-rich tumors, offering a safe and effective strategy for solid tumor treatment.

### 4.3 YAP/TAZ inhibitors

Combined use of YAP/TAZ inhibitors may disrupt physical barriers and amplify antitumor immune responses. Current strategies focus on inducing YAP/TAZ protein degradation, interfering with YAP/TAZ-TEAD transcription factor binding, blocking YAP/TAZ nuclear localization, or targeting upstream regulatory pathways. First-generation YAP/TAZ inhibitors, such as verteporfin, aim to block YAP/TAZ-TEAD 1–4 interactions but exhibit off-target effects on autophagy and TNF signaling (109). Next-generation TEAD inhibitors competitively bind to the conserved hydrophobic palmitate-binding pocket of TEAD proteins, inhibiting palmitoylation and destabilizing the YAP/TAZ-TEAD complex. Antisense oligonucleotides (e.g., ION537) degrade YAP mRNA to disrupt YAP/TAZ-TEAD interactions (110). YAP/TAZ inhibitors may also suppress CAF activity, reduce ECM deposition, and improve neoantigen-specific TIL infiltration.

### 4.4 Hyaluronic acid degradation

Excessive accumulation of high-molecular-weight hyaluronic acid (HA) in tumor ECM forms a dense gel-like structure, increasing IFP and impeding immune cell penetration. Hyaluronidase-mediated HA degradation reduces tumor ECM stiffness. PEGylated human hyaluronidase (PEGPH20) enzymatically depletes intratumoral HA, as shown in preclinical and clinical studies (111). For example, PEGPH20 enhances therapeutic efficacy in pancreatic cancer models by degrading HA (112). HA degradation may reduce ECM stiffness, alleviate interstitial pressure, and promote neoantigen-specific TIL migration. However, excessive HA degradation risks releasing tumor cells and facilitating metastasis. Thus, precise control of HA degradation is critical to minimize adverse effects and maximize neoantigen therapy efficacy (113).

### 4.5 Tumor vasculature normalization

Anti-angiogenic drugs transiently “normalize” tumor vasculature by “starving” tumors, improving perfusion, reducing hypoxia, and enabling neoantigen-specific TILs to reach tumor sites. VEGF stimulates abnormal tumor vasculature, impairing neoantigen-specific TIL function. The anti-VEGF monoclonal antibody bevacizumab binds VEGFA, blocking its interaction with VEGFR-2 on endothelial cells, progenitor cells, and megakaryocytes. Bevacizumab also suppresses Treg proliferation in metastatic colorectal cancer patients (114). Anti-VEGF monotherapy may transiently normalize the vasculature and improve neoantigen-specific TIL infiltration, but prolonged use risks exacerbating hypoxia. Bevacizumab also faces challenges such as drug resistance, toxicity, and the potential for short-term treatment to enhance tumor aggressiveness. Therefore, identifying strategies to overcome anti-VEGF resistance is critical. The angiopoietin family (Angiopoietins) includes Ang1 and Ang2. Ang1, secreted by pericytes, maintains vascular integrity by activating the Tie2 receptor, stabilizing vascular structure (115). Ang2, stored in Weibel-Palade bodies by endothelial cells, promotes vascular destabilization and neovascularization (116). Endothelial cell responsiveness to Ang1 depends on the relative levels of its receptor Tie2 and the inhibitory co-receptor Tie1. Tie1 interacts with Tie2 on the cell surface, suppressing Ang1-Tie2 signaling. Regulated cleavage of Tie1—activated by VEGF, inflammatory factors, and shear stress—relieves inhibition of Tie2, enhancing Ang1 signaling (117). The Angiopoietin-Tie pathway plays a pivotal role in tumor angiogenesis by regulating vascular stability, remodeling, and microenvironment interactions. AMG-386 inhibits angiogenesis by blocking Ang1/2 binding to Tie2. Combining angiopoietin inhibitors, particularly Ang2-targeted agents, with VEGF inhibitors effectively addresses resistance to single-target therapies (107, 116). By suppressing the tumor vascular barrier’ s impact on neoantigen-specific TILs, this approach further optimizes the clinical efficacy of neoantigen-based therapies.

### 4.6 Other therapeutic strategies

Therapeutic strategies targeting other ECM components, such as fibronectin and laminin, primarily focus on inhibiting their abnormal deposition or blocking their tumor-promoting signaling (118). For fibronectin, targeting its pro-fibrotic EDA isoform can suppress CAF secretion, thereby reducing ECM stiffness-induced obstruction of neoantigen-specific TILs (119). Researchers have also engineered Salmonella to continuously secrete VNPNKase, degrading fibronectin and inhibiting CAF fibrosis (120). For laminin, strategies involve inhibiting interactions between its key isoforms (e.g., LM-111, LM-332) and the tumor microenvironment (121). These approaches aim to reduce ECM density and reverse its immunosuppressive effects, enhancing TIL infiltration and function. Beyond ECM-targeted strategies, chemokine receptor antagonists (e.g., CXCR1/2 inhibitors) can disrupt chemokine-mediated barriers by reducing pro-tumorigenic neutrophils (122). Physical therapies such as photothermal, ultrasound, and laser treatments also address challenges in neoantigen therapy. Photothermal agents convert light energy into heat, elevating local temperatures to destroy tumor cells, soften/densegrade ECM, lower IFP, and promote neoantigen-specific TIL infiltration (123). Novel delivery systems show promise in improving TIL penetration. Nanoparticle delivery systems enable precise anticancer drug targeting by mimicking natural metabolic pathways and camouflaging drug structures (124). They reduce off-target biodistribution of active pharmaceutical ingredients, significantly minimizing treatment side effects (125). Leveraging tumor hyperpermeability and poor lymphatic drainage, nanoparticles selectively accumulate in tumors. Future efforts may focus on combining neoantigen therapy with nanoparticle systems to modulate tumor biomechanics, enhance TIL cytotoxicity, and achieve efficient anticancer precision medicine (126).

## 5 Conclusion

The TME hinders the infiltration of neoantigen-specific TILs into the tumor core by forming physical barriers. Therefore, studying these physical barriers may provide novel strategies to overcome the limitations of neoantigen-based immunotherapy. This article discusses the physical barriers faced by neoantigen-specific TILs, aiming to elucidate the multidimensional mechanisms by which physical factors influence their function. Tumor mechanical stress, generated during tumor growth due to cell proliferation, ECM stiffening, and altered interstitial pressure, impacts neoantigen-specific TILs by modulating tumor cell behavior and the surrounding microenvironment (127). These mechanical stresses include fluid shear stress and solid stress. Fluid shear stress arises from interstitial fluid flow or blood circulation within the TME, while solid stress results from tumor cell proliferation, ECM stiffening, and compression by surrounding tissues (128). Tumor cells and CAFs secrete collagen and fibronectin, leading to ECM crosslinking and stiffening. Mechanical stresses (e.g., matrix stiffness) activate integrin signaling pathways (e.g., FAK, Rho/ROCK) and mechanosensitive transcription factors (e.g., YAP/TAZ), further remodeling the ECM (129, 130). Abnormal tumor angiogenesis generates fluid shear stress that disrupts vascular endothelial cell function, promoting vascular leakage and inflammatory cytokine release (131). Vascular leakage, impaired lymphatic drainage, ECM densification, and high cellular metabolism collectively cause interstitial fluid accumulation, creating a high-pressure environment. ECM stiffening interacts with elevated IFP to directly compress blood and lymphatic vessels, obstructing fluid drainage. High IFP compresses blood vessels, limiting the migration and infiltration of neoantigen-specific TILs. Tumor mechanical stress regulates ECM stiffening, aberrant angiogenesis, and high IFP, creating a vicious cycle that forms a composite barrier with both spatial obstruction and immunosuppressive properties, ultimately impairing neoantigen-specific TILs.

The clinical translation of neoantigen-specific TILs is limited by the complex regulatory network of tumor physical barriers. Current clinical trials targeting these physical barriers remain in early exploratory stages. Existing trials primarily focus on evaluating the efficacy and safety of combining neoantigens with immune checkpoint inhibitors, radiotherapy, chemotherapy, and targeted therapies, while lacking synergistic regulation of ECM stiffness, vascular abnormalities, and mechanical stress signaling pathways. Interventions such as LOX inhibitors and hyaluronidase (HA)-degrading enzymes can improve local stromal permeability but carry risks of promoting metastasis and face contradictions in therapeutic timeliness, highlighting the need to develop multimodal synergistic intervention strategies. Future efforts should advance multiscale biomechanical regulation systems, explore the combination of physical modulators with neoantigen vaccines, and leverage mechanical interventions to enhance neoantigen-specific TIL infiltration. Additionally, integrating novel delivery systems with neoantigen vaccines may emerge as a key technology to improve neoantigen-specific TILs. Establishing interdisciplinary medical platforms that integrate biomechanics and immunotherapy, along with in-depth analysis of the interplay between physical barriers and immune metabolism, will provide novel paradigms for solid tumor immunotherapy.
